# Expression and function of epithelial cell adhesion molecule EpCAM: where are we after 40 years?

**DOI:** 10.1007/s10555-020-09898-3

**Published:** 2020-06-07

**Authors:** Olivier Gires, Min Pan, Henrik Schinke, Martin Canis, Patrick A. Baeuerle

**Affiliations:** 1 Department of Otorhinolaryngology, University Hospital, LMU Munich, Marchioninistr. 15, 81377 Munich, Germany; 2 Clinical Cooperation Group “Personalized Radiotherapy in Head and Neck Cancer”, Helmholtz Zentrum, Neuherberg, Germany; 3 Department of Otorhinolaryngology, The First Affiliated Hospital of Chongqing Medical University, No. 1 Youyi Road, Yuzhong District, Chongqing, 400016 China; 4 Institute for Immunology, LMU Munich, Grosshadernerstr. 9, 82152 Planegg, Martinsried, Germany; 5 MPM Capital, Cambridge MA, 450 Kendall Street, Cambridge, MA 02142 USA

**Keywords:** EpCAM, Carcinoma, Metastasis, Regulated intramembrane proteolysis, Liquid biopsy, Epithelial-to-mesenchymal transition

## Abstract

EpCAM (epithelial cell adhesion molecule) was discovered four decades ago as a tumor antigen on colorectal carcinomas. Owing to its frequent and high expression on carcinomas and their metastases, EpCAM serves as a prognostic marker, a therapeutic target, and an anchor molecule on circulating and disseminated tumor cells (CTCs/DTCs), which are considered the major source for metastatic cancer cells. Today, EpCAM is reckoned as a multi-functional transmembrane protein involved in the regulation of cell adhesion, proliferation, migration, stemness, and epithelial-to-mesenchymal transition (EMT) of carcinoma cells. To fulfill these functions, EpCAM is instrumental in intra- and intercellular signaling as a full-length molecule and following regulated intramembrane proteolysis, generating functionally active extra- and intracellular fragments. Intact EpCAM and its proteolytic fragments interact with claudins, CD44, E-cadherin, epidermal growth factor receptor (EGFR), and intracellular signaling components of the WNT and Ras/Raf pathways, respectively. This plethora of functions contributes to shaping intratumor heterogeneity and partial EMT, which are major determinants of the clinical outcome of carcinoma patients. EpCAM represents a marker for the epithelial status of primary and systemic tumor cells and emerges as a measure for the metastatic capacity of CTCs. Consequentially, EpCAM has reclaimed potential as a prognostic marker and target on primary and systemic tumor cells.

## Introduction

The epithelial cell adhesion molecule (EpCAM) has first been described in 1979 as a humoral antigen expressed on colon carcinoma cells [1]. Here, we have summarized the impressive progress in the biology of EpCAM in the past four decades. Expression patterns, regulation, and the multiple functions of EpCAM in normal epithelia, in carcinoma, and in pluripotent stem cells are reviewed. Furthermore, the clinical implications and applications related to EpCAM are discussed. Lastly, the most recently discovered involvement of EpCAM in the regulation of epithelial-to-mesenchymal transition, which is of paramount importance during metastases formation and therapy resistance, is delineated.

### Initial description

**Fig. 1:**
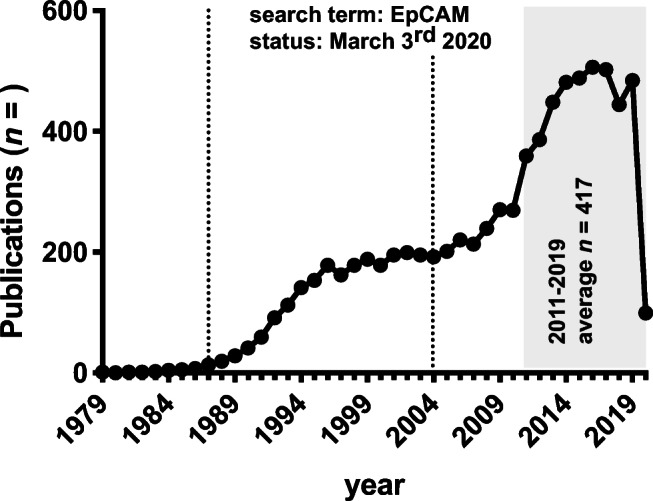
Publication numbers retrieved from PubMed using “EpCAM” as a search term. Two time points of increase in publication numbers are marked by dashed lines. The first wave of increased publications maps to the cloning of the EpCAM cDNA in 1989. The second wave coincides with the description of EpCAM function in proliferation and migration in 2004

EpCAM was first described in 1979 as a humoral antigen recognized by monoclonal antibody 1083-17-1A (Co17-1A or mAb 17-1A) following inoculation of human colorectal cancer cells in mice [1]. Today, PubMed-listed publications on “EpCAM” exceed 8000 entries. Publications have steadily increased (Fig. 1) reflecting an enhanced interest in EpCAM along with broadening functional roles.

### EpCAM cDNA cloning and protein structure

The coding sequence of human EpCAM was deciphered in 1989 and predicted a single transmembrane protein of 314 amino acids (aa) with a 265-aa extracellular domain, a 23-aa hydrophobic transmembrane domain, and a hydrophilic 26-aa intracellular domain [2]. In 2014, the crystal structure of the extracellular domain of EpCAM was resolved and was shown to represent heart-shaped homodimers [3].

### Cell adhesion

The first function as a homophilic cell adhesion molecule and a role in the integrity of epithelium was proposed in conjunction with the name EpCAM by Litvinov et al. [4]. They reported that ectopic expression of EpCAM in murine fibroblasts and mouse mammary carcinoma cells induced clustering and segregation of cells and reduced invasive growth. Later on, inhibitory activity towards cadherin-mediated adhesion in epithelial cells was reported [5].

### Proliferation and differentiation

First evidence for a correlation of EpCAM in proliferation and differentiation surfaced in 1994 [6] and 1996 [7] in keratinocytes, transformed epithelial cells, and carcinoma cell lines. In 2004, a role for EpCAM in the regulation of proliferation, migration, and invasion was shown [8, 9]. A role of EpCAM in differentiation was reported in pluripotent embryonic stem cells (ESCs), progenitor cells, and carcinoma stem cells [10].

### Clinical significance in cancer

Frequent and high-level expression of EpCAM on various carcinomas (98 out of 131 tested) [11, 12] and metastases [13, 14], and a correlation with clinical outcome qualified it as prognostic marker and therapeutic target [15]. EpCAM-specific antibody Panorex® (edrecolomab; 17-1A) first attained market approval for treating colorectal carcinomas in 1995 [16]. Furthermore, EpCAM served to enrich, identify, and characterize metastatic cells that have disseminated from primary tumor into blood and bone marrow of advanced carcinoma patients [17, 18]. Despite existing challenges, EpCAM remains the surface antigen of choice in clinical use to isolate circulating tumor cells (CTCs) with prognostic value and metastatic potential [15, 19–21].

### EpCAM in non-malignant diseases

A major breakthrough in understanding congenital tufting enteropathy (CTE), a severe form of early-onset autosomal recessive diarrhea, was achieved when Mamata Sivagnanam and colleagues reported on the linkage of CTE with mutations in the *EPCAM* gene that precluded its correct expression at the plasma membrane [22]. Lack of EpCAM expression results in villus atrophy and in the formation of intestinal tufts, which eventually induces a dysfunctional intestinal barrier and unbalanced ion transport [23, 24].

Furthermore, mutations in the 3′-end of the *EPCAM* gene induce epigenetic silencing of genes downstream of *EPCAM* that are involved in mismatch repair, including the MutL homolog 1 (*MLH1*) and MutS protein homolog 2 (*MSH2*) genes. *EPCAM* 3′-mutations and subsequent deregulation of MLH1 and MSH2 protein expression are the cause of Lynch syndrome (hereditary non-polyposis colorectal cancer (HNPCC)) [25, 26].

**Fig. 2:**
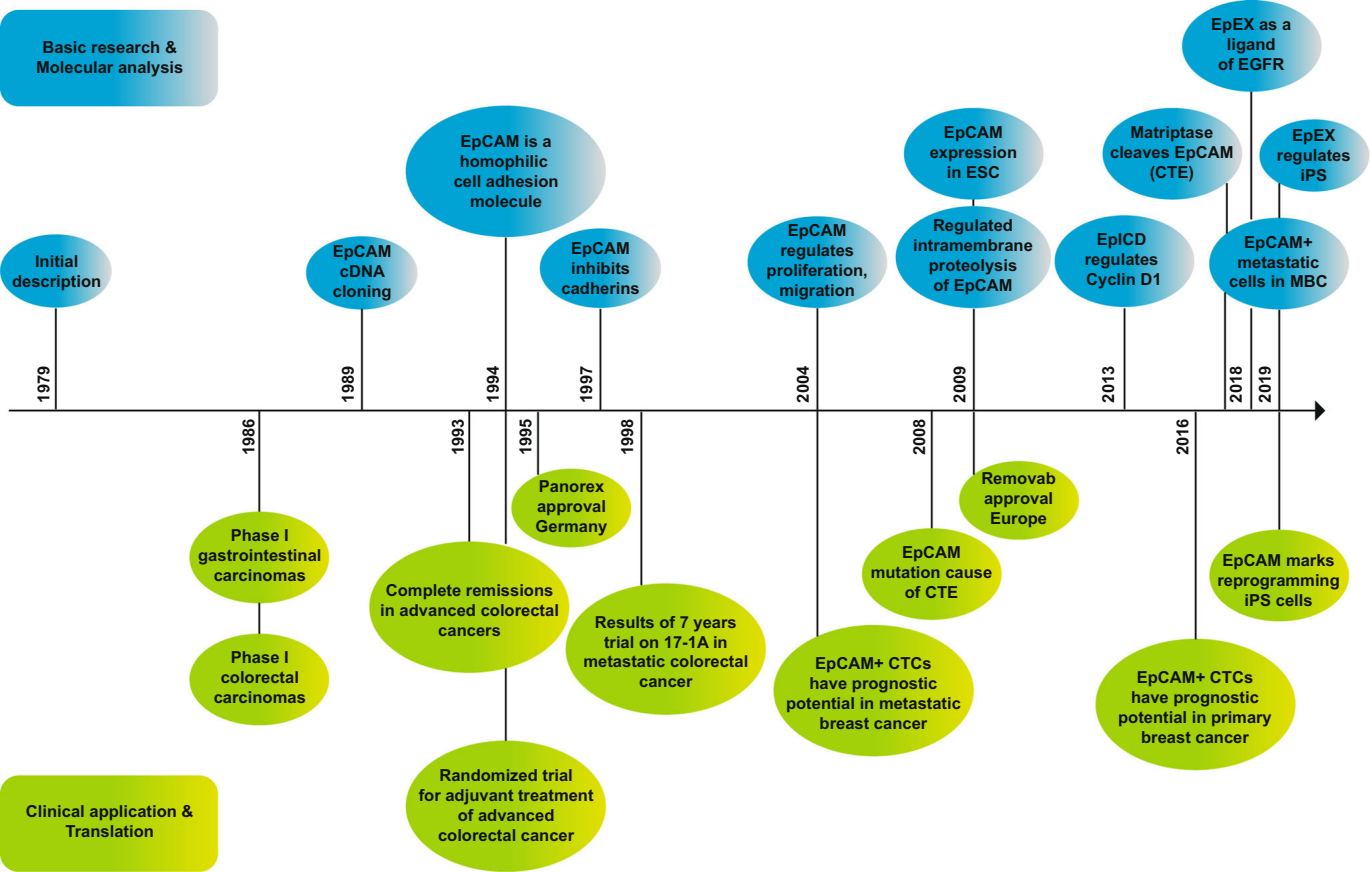
Milestones of EpCAM discoveries in basic research (in blue) and in clinical application (in green). ESC: embryonic stem cells, CTE: congenic tufting enteropathy, iPS: induced pluripotent stem cells, MBC: metastatic breast cancer, CTCs: circulating tumor cells

A chronic of major advances on EpCAM in basic research and clinical application is summarized in Fig. 2.

## EpCAM gene and protein structure

The human *EPCAM* gene is encoded on the plus strand of chromosome 2p21 and consists of 9 exons covering 41.88 kilobases (kb). Exon 1 encodes the 5′-untranslated region and the signal peptide, exon 2 the EGF-like motif, exon 3 the thyroglobulin domain, exons 4–6 the cysteine-poor part of the domain, exon 7 the transmembrane domain, exon 8 parts of the intracellular domain, and exon 9 the remaining intracellular domain and the 3′-untranslated region [27]. A 1.1-kb fragment of the *EPCAM* promoter sufficient to drive gene expression and confers epithelial specificity was cloned [28, 29]. The promoter can be further subdivided in a gene proximal part composed of 570 base pairs (bp) and a distal part of 550 bp that act synergistically in *EPCAM* expression and are negatively regulated by nuclear factor kappa B (NF-κB) [29]. Sankpal et al. further described the usage of an extracellular-regulated kinase 2 (ERK2) binding site within the *EPCAM* promoter [30], while Yamashita et al. reported on the regulation of the *EPCAM* promoter by a Wnt-β-catenin-Tcf4 complex in hepatocellular carcinoma cells [31]. Furthermore, the EMT-inducing transcription factor Zeb1 represses *EPCAM* expression in zebrafish [32].

**Fig. 3:**
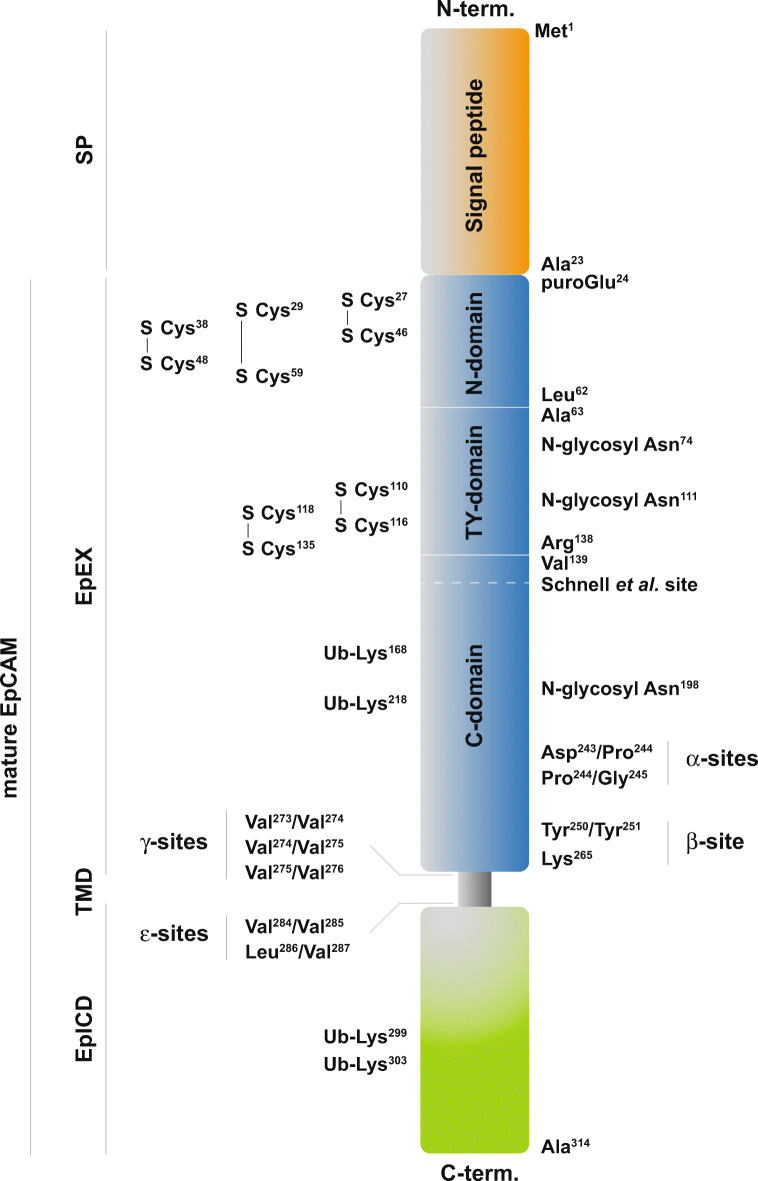
Schematic representation of the EpCAM protein. EpCAM is composed of a signal peptide (SP) that is removed from the mature protein. Mature EpCAM comprises an extracellular domain (EpEX), a single transmembrane domain (TMD), and a short intracellular domain (EpICD). N-Terminal (N-domain), thyroglobulin (TY-domain), and C-terminal domains (C-domain) within EpEX, as defined by Pavsic et al. [3], are marked. N- and TY-domains are cysteine-rich protein stretches that have initially been defined as EGF-like domains. Disulfide bonds involving cysteines, N-glycosylation at asparagines, ubiquitylation at lysines, and cleavage sites related to regulated intramembrane proteolysis of EpCAM (α-, β-, γ-, and ε-sites) [33, 34] are annotated. The approximated additional cleavage site reported by Schnell et al. [35] is indicated. Sizes are not at scale

EpCAM is a transmembrane protein with a single membrane-spanning domain (23-aa) that connects the larger extracellular domain (265-aa) to a short intracellular domain (26-aa) (Fig. 3). The extracellular domain contains a signal peptide, an EGF-like, cysteine-rich domain, and a thyroglobulin-like domain, which was initially referred to as a second EGF-like repeat [36], followed by a cysteine-poor region [37]. Mass spectrometry and Edman sequencing of the extracellular domain of EpCAM demonstrated the cleavage of the signal peptide after aa 23, resulting in an N-terminus starting with a modified pyroglutamate [38]. Disulfide bonds were mapped to Cys^27^–Cys^46^, Cys^29^–Cys^59^, Cys^38^–Cys^48^, Cys^110^–Cys^116^, and Cys^118^–Cys^135^ (Fig. 3) [38].

N-Glycosylation of EpCAM has been reported with no evidence of O-glycosylation [2]. N-Glycosylation sites have been mapped to Asn^74^, Asn^111^, and Asn^198^ of EpCAM [38]. Initially complete glycosylation of Asn^111^, partial glycosylation of Asn^74^, and no glycosylation at Asn^198^ were reported [38]. However, single and dual mutations of Asn^74^ and Asn^111^ revealed that mutations retained a certain degree of glycosylation, which was lost when all positions including Asn^198^ were mutated [39]. Loss of glycosylation at this site resulted in severe reduction of protein stability and half-life at the membrane from 21 to 7 h [39]. Glycosylation of EpCAM may impact on EpCAM stability and expression in tumor cells, as it was found increased in cancerous *versus* healthy tissue [40].

Crystal structure analysis of the extracellular domain of EpCAM, termed EpEX, at 1.86 Å resolution revealed a three-partite fold in amino-terminal (puroGlu^24^–Leu^62^), thyroglobulin-like (Ala^63^–Arg^138^), and carboxy-terminal (Val^139^–Lys^265^) domains (Fig. 3). The respective ND, TY, and CD domains are each in contact with the other two domains, forming a triangle [3]. EpEX molecules form *cis*-dimers with strongest interactions between the TY loop of one EpEX molecule with the βC sheet of a second molecule [3]. Coarse grain modelling of the intramembrane domain demonstrated a dimeric structure in which two helices of the membrane-spanning parts are symmetrically arranged and cross each other between Val^276^ and Val^280^ [3]. There is so far no structural information of the intracellular domain of EpCAM termed EpICD.

## EpCAM and cell adhesion

Initial characterization of EpCAM functions was conducted in murine fibroblasts and L153S mammary carcinoma cells, which are characterized by loss of cell adhesion and the adoption of a spindle-shaped morphology. Ectopic expression resulted in increased intercellular adhesion and cell aggregation in suspension, along with the segregation of EpCAM-positive and EpCAM-negative cells, and a reduced capacity of fibroblast to grow invasively [4, 41]. Membrane-proximal thyroglobulin-like domains, initially referred to as a second EGF-like domain, mediate lateral interactions of EpCAM in *cis* on one cell. Membrane-distal EGF-like repeats are required for interactions of EpCAM in *trans* on adjacent cells [42]. Taken together, formation of functional EpCAM tetramers as the initiating event in the formation of cell adhesion complexes was proposed [42].

Surprisingly, overexpression of EpCAM in epithelial cell lines that depend on cadherin-mediated cell-cell connections decreased adhesion by impairing functional *adherens* junctions through disruption of E-cadherin, α-catenin, and F-actin interactions [43, 44]. EpCAM’s capacity to inhibit cadherin-mediated adhesion in breast epithelial cells depended on phosphoinositide 3-kinase (PI3K) and its shifted interaction with N-cadherin to EpCAM [45].

Knockout of EpCAM in mice supported its role in orchestrating the structure and functionality of epithelium in the intestinal tract [46–48]. EpCAM knockout induced an abnormal placental development associated with death *in utero* at gestation day E12.5 [46]. Subsequent knockouts did not reproduce an embryonic lethality; however, mice died of severe intestinal erosion and hemorrhagic diarrhea shortly after birth [44, 45]. Intestinal defects were reminiscent of human CTE where mutations cause a loss of EpCAM expression at the plasma membrane [22, 49]. Despite agreeing on the observed phenotype, Lei et al. [48] and Guerra et al. [47] disagreed on the molecular mechanisms. Lei et al. observed a loss of tight junction formation owing to substantially reduced recruitment of murine claudin 7 to tight junctions in the absence of EpCAM [48]. These findings are supported by the direct interaction of EpCAM with claudin 7 [50], which was demonstrated to support tumor progression in colorectal cancer [51, 52]. Guerra et al. demonstrated a dysregulation of E-cadherin and ß-catenin functions leading to partial disruption of *adherens* junctions [47]. A cooperation of E-cadherin and EpCAM in epithelial adhesion regulation is further supported by work in zebrafish, where EpCAM knockout impaired the integrity of the skin periderm through reduced cell surface levels of E-cadherin and increased levels of tight junction protein 1 (Tjp1) [53].

More discrepancies exist on EpCAM’s role in cell-cell adhesion. Gaber et al. [54] could not confirm intercellular homo-oligomers, despite formation of *cis*-dimers [54], leading to the conclusion that EpCAM’s role in adhesion is not assumed as a homophilic cell adhesion molecule. Furthermore, neither regulated intramembrane proteolysis of EpCAM nor *EPCAM* knockout in cell lines had any measurable impact on cell-matrix and cell-cell adhesion in cancer cells [33]. Hence, EpCAM’s molecular function in adhesion has not been satisfactorily resolved yet. EpCAM is undeniably involved in maintenance of epithelial integrity in various animal models and human conditions where it acts in concert with established cell adhesion molecules. Therefore, EpCAM might support cell adhesion primarily mediated by other molecules such as claudins and cadherins, and/or might preferentially play a role in adhesion of normal but not tumor cells.

## EpCAM as a prognostic marker in cancer

**Fig. 4:**
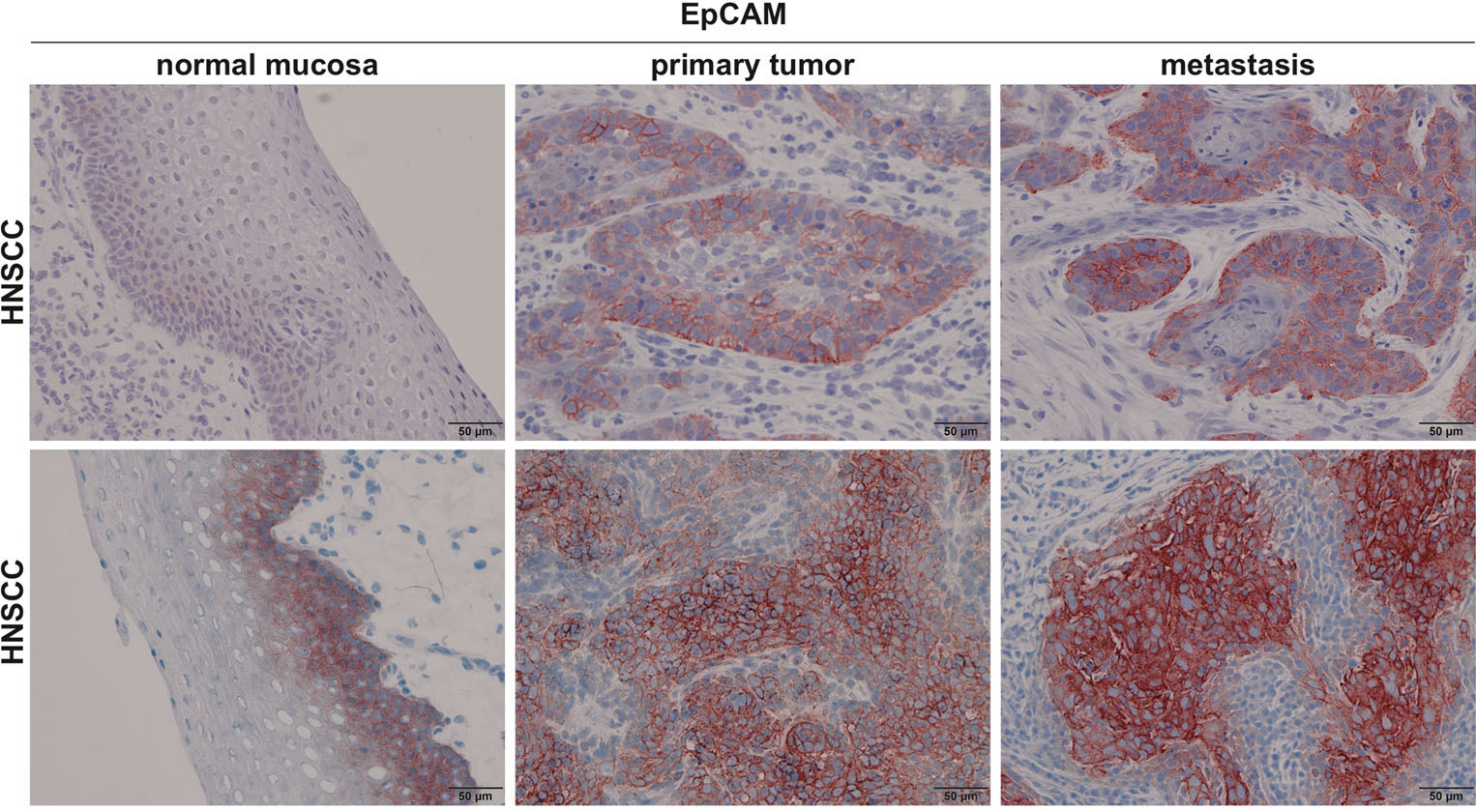
EpCAM expression in normal mucosa, primary head and neck squamous cell carcinoma, and lymph node metastasis. Shown are immunohistochemistry staining of EpCAM in normal mucosa, primary tumor, and lymph node metastases of head and neck squamous cell carcinomas (HNSCC)

EpCAM received considerable attention as a prognostic marker based on its strong expression in various carcinomas and their metastases as compared to normal epithelia of the same localization [11–13]. EpCAM is highly and frequently expressed in the vast majority of carcinomas that have been analyzed [11, 12] and its expression in metastases frequently correlates with levels in the corresponding primary tumors (Fig. 4 and [13, 14, 21, 55]). As such, EpCAM bears potential as a prognostic and therapeutic marker in carcinomas and during cancer progression and metastasis formation. However, EpCAM’s prognostic value varies depending on the tumor entity. High expression in primary carcinomas is associated with poor prognosis in breast [56, 57], colorectal [58], prostate [59], gallbladder [60], ovarian [61], bladder [62], pancreas [63, 64], and adenoid cystic carcinomas [65]. Oppositely, high expression of EpCAM is associated with better prognosis in colonic [12], esophageal [66], renal [67, 68], gastric [69], endometrial [70], thyroid [71], and head and neck carcinomas [72]. Multiple cellular functions of EpCAM may deploy in dependency of tumor types and localization, and might differently affect single cells within tumors. High expression of EpCAM can promote sustained proliferation and tumor/metastatic growth and might thereby be associated with poor prognosis. EpCAM also represents a marker of epithelial differentiation and might therefore associate with a more differentiated, less migratory/invasive phenotypes, with reduced resistance to irradiation and chemotherapy, and hence with better survival. Therefore, EpCAM’s association with differential clinical outcome is complex and might vary depending on the origin of the tumor or even the stage of tumor progression. This unsolved discrepancy in the prognostic value of EpCAM expression remains poorly understood and requires further investigation.

Another essential aspect is that cancer-related lethality is primarily caused by therapy-resistant cells and frequently untreatable metastases. Numerous studies on EpCAM’s prognostic value have initially concentrated on primary tumors rather than disseminated cells [12, 57–65, 67–73]. However, primary tumor biopsies represent a specific region of the tumor at a given time point. Based on growing evidence of intratumor heterogeneity and temporal variation in tumor antigen expression [74–80], a singular measurement of primary tumors might suffer from bias. Consequently, numbers of disseminated tumor cells, which are considered primary sources of relapse and metastases, have been implemented as additional prognostic marker [81–83]. Here, tumor cells found in the blood are termed circulating tumor cells (CTCs) and tumor cells in distant organs, for example, in the bone marrow, are termed disseminated tumor cells (DTCs). Clinical assessment of CTCs in the blood is minimally invasive and allows for longitudinal measurements [82, 84] and aims at the prediction of metastasis formation [19]. Measurement of CTCs in the blood of metastatic breast cancer (MCB) patients was performed using EpCAM-specific antibodies for the enrichment of rare circulating tumor cells, which were subsequently further characterized by the expression of cytokeratins, presence of a nucleus, and lack of white blood cell marker CD45. CTC numbers in the blood showed prognostic value and patients with five CTCs or more *per* 7.5 mL blood had decreased overall and progression-free survival [18]. More recently, 3173 patients with non-metastatic (stages I–III) breast cancer were analyzed and a CTC threshold of ≥ 1 cell per 7.5 mL of blood correlated with decreased OS, disease-free survival, distant disease-free survival, and cancer-specific survival [85]. The prognostic value of EpCAM-positive CTCs has been confirmed for further tumor entities, including lung cancer [86], advanced ovarian cancer [87], gastric cancer [88], colorectal cancer [89], and head and neck squamous cell carcinoma (HNSCC) [90]. Hence, the enrichment of rare EpCAM-positive systemic tumor cells in the blood in the frame of liquid biopsies has the potential to evaluate disease outcome including the patients’ burden of metastatic cells [20, 21, 85].

## EpCAM as a target in clinical trials for cancer therapy

EpCAM is highly and frequently expressed in the vast majority of carcinomas, in tumor-initiating cells, and in disseminated tumor cells, which qualified EpCAM as a potential target for cancer therapies [15, 91, 92]. Several publications demonstrated that EpCAM-specific antibodies can eliminate cancer cells by various mechanisms [93–102] and detect DTC in the bone marrow of patients [103, 104]. Monoclonal antibody 17-1A was clinically tested alone and in combination with γ-interferon for the treatment of gastrointestinal and metastasized colorectal carcinomas [105, 106]. Induction of complete remissions by mAb 17-1A were reported in metastasized colorectal cancer [107, 108]. Positive overall survival data with mAb 17-1A in a pivotal study with metastasized colorectal cancer patients [96, 99] resulted in approval of the antibody in Germany under the trade name Panorex®. However, when compared to standard care with 5-fluorouracil-based chemotherapy in a later trial [109], edrecolomab was found inferior, leading to its market withdrawal. EpCAM-specific humanized antibodies ING-1 and 3622W94, bearing higher binding affinity than edrecolomab, were tested in clinical trials but were discontinued because of low tolerability. While edrecolomab might have suffered from an insufficient binding affinity, ING-1 and 3622W94 displayed too high affinity that no longer allowed to distinguish normal and malignant cells, leading to pancreatitis [110, 111].

Micromet Inc. developed fully human IgG1 EpCAM-specific antibody MT201 (adecatumumab) that had an intermediate binding affinity for EpCAM in an attempt to improve therapeutic efficacy [112]. Adecatumumab was investigated as monotherapy in a phase II clinical trial in MBC patients. Adecatumumab did not induce measurable tumor regression; however, patients treated with high-dose antibody and expressing high levels of EpCAM retrospectively showed reduced development of new metastases (3/18 *versus* 14/29 patients) [113]. In a dose-escalating phase I trial enrolling prostate cancer patients, reduction of prostate-specific antigen levels was observed [114]. While having a favorable safety profile, the antibody program is no longer pursued because it did not reach its clinical endpoints.

EpCAM was also targeted with T cell-engaging antibodies designed to connect cytotoxic T cells with cancer cells for redirected lysis. Antibody catumaxomab (Removab®) developed by Trion Pharma has an EpCAM- and a CD3-binding arm and its Fc domain binds Fc gamma-receptor expressing antigen-presenting cells [115]. Catumaxomab was approved by the European Medicines Agency (EMA) to treat malignant ascites [116], but was eventually terminated. MT110 (solitomab) is a tandem single-chain antibody construct comprised of an EpCAM- and CD3-specific binding domain developed by Micromet Inc. [117]. Pharmacokinetics, tolerability, safety, and anti-tumor activity of MT110 were assessed in a dose-escalating phase I clinical trial in metastatic colorectal, gastric, and lung carcinomas (129) with disease stabilization in 7/19 patients. Due to gastrointestinal toxicities, solitomab could not be escalated to more efficacious dose levels, and the program was abandoned.

Recently, EpCAM-specific antibody EpAb2-6 was developed, which binds to an epitope within the thyroglobulin domain and demonstrated inhibitory potential [118]. EpAb2-6 induces apoptosis, inhibits tumor growth in mouse models, and represses EpCAM cleavage to form the signaling moiety EpICD in pancreatic and colon carcinoma cells [118, 119]. Thus, EpAb2-6 is an anti-EpCAM antibody that inhibits central EpCAM signaling functions, which might represent a novel approach to treatment methods targeting EpCAM in carcinomas. Furthermore, EpCAM-specific antibodies were conjugated with bouganin (citatuzumab bogatox) [120], *Pseudomonas* exotoxin A (oportuzumab monatox) [121] and alpha-amanitin [122], with interleukin-2 to activate T cells in the vicinity of EpCAM-positive tumor cells (huKS-IL2) [123], and with encapsulated inhibitory RNAs [124–126]. In 2015, a phase I clinical trial of patients with EpCAM-positive metastatic cancers aimed at evaluating the maximum tolerated doses, pharmacokinetics, and immunogenicity of the anti-EpCAM-based immunotoxin MOC31PE that is coupled to *Pseudomonas* exotoxin A. MOC31PE was intravenously combined with the immunosuppressant cyclosporin in *n* = 63 metastatic patients and revealed a safe profile with a half-life in plasma of 3 h and a reduction of [127]. Subsequently, MOC31PE was applied to address peritoneal metastasis in colorectal and ovarian cancers within the ImmunoPeCa phase I/II. Based on the promising cytotoxicity profile and a low systemic uptake, MOC31PE is currently further evaluated in a clinical phase II study [128, 129].

Further clinical trials including the usage of chimeric antigen receptor T cells (CAR T cells) targeting EpCAM are ongoing in order to address their potential for the treatment of recurrent and treatment-resistant solid tumors (https://clinicaltrials.gov/ct2/show/NCT04151186). Currently, a total of 64 clinical trials are listed by the US national library of medicine that involve EpCAM as a biological using CAR T cells, monoclonal antibodies, immunotoxins and immunocytokines, and EpCAM-based capture methods to enrich CTCs (https://clinicaltrials.gov/ct2/results?cond=&term=EpCAM&cntry=&state=&city=&dist=). While EpCAM expression on most carcinoma is a highly attractive feature for tumor-associated antigens, expression on healthy epithelia—mostly of the gastrointestinal tract—will limit the therapeutic window of EpCAM-targeted therapies and call for potential side effects. A next generation of EpCAM-targeted drugs that are selectively activated in the tumor microenvironment may finally allow to leverage this target antigen [130].

Finally, EpCAM serves as a target for image-guided surgery approaches that aim at improving resection margins. Residual tumor cells following incomplete tumor removal at the resection margin are a major source of local recurrence in solid tumors [131]. The employed strategies include the systemic application of EpCAM-specific antibodies labeled with fluorescent dyes such as fluorescein isothiocyanate (FITC) and near-infrared fluorescence dye (NIRF) IRDye800CW [132, 133]. Fluorescence-labeled anti-EpCAM antibodies allow the detection of residual tumor nodules in the millimeter size range using intraoperative imaging systems [133, 134].

## Signaling of EpCAM

### RIP-mediated EpCAM signaling

**Fig. 5:**
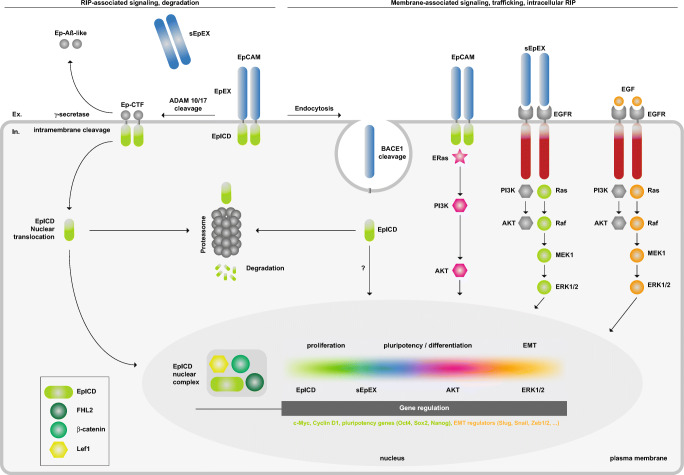
Schematic representation of the signaling mechanisms associated with EpCAM. Signaling by EpCAM and degradation of EpCAM *via* regulated intramembrane proteolysis (RIP) is depicted in the left part of the scheme. Membrane-associated signaling, trafficking, and intracellular RIP in endocytic vesicles are depicted on the right part. Effects of distinct pathways and the associated molecules are color-coded and implemented in the schematic representation of the cell nucleus

EpCAM regulates cell cycle progression and differentiation *via* regulated intramembrane proteolysis (RIP). Initial cleavage of EpCAM is conducted by membrane-resident ADAM (a disintegrin and metalloproteinase) family proteases ADAM 10 and 17 (Fig. 5). Thereby, the ectodomain of EpCAM is shed into the extracellular space as soluble EpEX [135]. The resulting membrane-tethered C-terminal fragment (EpCAM-CTF) is cleaved by the γ-secretase complex to form the extracellular, small, and soluble Aβ-like fragment and the intracellular EpICD fragment [33, 34, 135]. EpICD translocates into the nucleus and, in combination with transcription factors and adaptor molecules such as FHL2, β-catenin, and Lef1, binds to promoter regions of regulators of cell division (cyclin D1 [136]), pluripotency genes [137–139], and genes involved in the regulation of EMT-associated processes such as tight junctions, adherence, and cell migration [140]. However, regulation of EpCAM cleavage and EpICD signaling through activation of EGFR remains a matter of dispute [140–143].

Interactions between ADAM 10 and EpCAM in the tetraspanin web of human colon cancer cells were reported before EpCAM RIP [144]. A role for ADAM 17 (tumor necrosis factor-α-converting enzyme (TACE)) in EpCAM RIP was demonstrated using biochemical approaches and TACE inhibitors [135]. Chemical inhibition of ADAMs and TACE knockdown prevented the first cleavage and reduced EpCAM’s proliferative effects, which were restored through ectopic expression of EpICD [135, 136]. Cleavage sites for ADAM proteases (α-sites) were determined in human and mouse EpCAM [33, 34]. Murine EpCAM has one major α-site (serine^230^/lysin^231^), whereas human EpCAM has two (aspartate^243^/proline^244^ and proline^244^/glycine^245^) [33, 34]. Additionally, the aspartyl protease BACE1 (beta-site amyloid precursor protein-cleaving enzyme 1) cleaves human and murine EpCAM at position tyrosine^250^/tyrosine^251^ and tyrosine^251^/tyrosine^252^ (β-site), respectively (Fig. 3). BACE1 has an optimal catalytic activity at pH 4 [145] and is therefore active in acidified cytoplasmic vesicles [146]. Under acidic conditions, cleavage of EpCAM is considerably more efficient, and BACE1 overexpression resulted in enhanced release of EpEX [34]. Based on superpositions of cleavage sites on the crystal structure of human EpEX, α- and β-sites are masked within EpEX dimers [3, 33, 147]. Superior cleavage by BACE1 could thus be facilitated owing to the reported decomposition of EpEX dimers to monomers under acidic conditions [3, 147]. However, quantitative contributions of ADAM proteases and BACE1 to the cleavage of EpCAM varied with the cell type [34].

Cleavage of ECAM-CTF is completed by the γ-secretase complex comprising presenilin-2 as catalytic subunit [135] (Fig. 5). Cleavages between residues Val^273^/Val^274^, Val^274^/Val^275^, and Val^275^/Val^276^ in human EpCAM-CTF and between Ala^271^/Val^272^ and Val^274^/Val^275^ (γ-sites) in murine EpCAM-CTF generate EpCAM-Aβ-like fragments. Cleavages between residues Val^284^/Val^285^ and Leu^286^/Val^287^ in human EpCAM-CTF and between Val^285^/Val^286^, Leu^287^/Val^288^, Val^288^/Iso^289^, and Ser^290^/Thr^291^ (ε-sites) in murine EpCAM-CTF produce EpICD molecules [33, 34]. Initial observation of a preferential proteolysis in malignant *versus* healthy colon tissue [135] was corroborated by independent findings. Firstly, RIP of EpCAM was not observed in fetal or adult liver cells, nor was it involved in regulating proliferation of these cells [148]. Secondly, EpICD nuclear translocation in various carcinomas correlated with more aggressive phenotypes and poorer outcome [149–151]. Lastly, generation of EpICD by γ-secretase is a particularly slow process with a 50% turnover of EpCAM-CTF between 0.75 and 5.5 h [152], as shown for other γ-secretase substrates [153]. Furthermore, EpICD is a highly labile protein that is degraded by the proteasome [34, 152], which might be controlled by two ubiquitylation sites at lysine^299^ and lysine^303^ within EpICD [154]. Based on all these findings, we believe RIP of EpCAM is most probably involved in a lengthy signaling process rather than in producing a signaling spike accompanied by comprehensive degradation of EpCAM.

The existence of a membrane version of EpCAM that is lacking the intracellular domain group has been reported [63], which correlated with a more aggressive phenotype of colorectal and pancreatic cancers and with reduced overall survival of the afflicted patients [58, 63]. Alternative EpCAM mRNA splicing was reported; however, none of these differentially spliced mRNAs would encode the truncated version of EpCAM (https://www.ncbi.nlm.nih.gov/ieb/research/acembly/av.cgi?db=human&term=EPCAM&submit=Go). Alternatively, rhomboid proteases and members of the signal peptide peptidase-like proteases (SPPL3) can cleave their substrates directly within the transmembrane domain. Thus, rhomboids could produce an EpCAM variant devoid of EpICD, but these proteases have only been demonstrated to cleave type II membrane proteins, whereas EpCAM is a type I membrane protein. Also, cleavage by SPPL3 and rhomboids is expected to release the substrates’ extracellular domains owing to the reduced hydrophobicity and lack of retention in the plasma membrane. Schnell et al. reported on γ-secretase-independent cleavage of EpCAM-CTF variants by membrane-associated proteases [35], which theoretically could cleave EpCAM without EpEX shedding. They further described an additional EpCAM cleavage at two sites within the cysteine-poor region that can alternatively generate EpICD [35]. Alternatively, the ectodomain of EpCAM may remain membrane-associated through the interaction with a yet unknown membrane component or post-translational modification such as acylation, which have not yet been described.

More recently, cleavage of EpCAM within the extracellular domain by the membrane-tethered serine peptidase matriptase was described [155]. Matriptase was found to catalyze dibasic cleavage of EpCAM between arginines 80 and 81, reducing the interaction of EpCAM with claudin-7 inducing its destabilization. This event has the potential to contribute to the disruption of the epithelial integrity of mouse intestine in CTE [156, 157].

### Signaling by intact EpCAM

Intact EpCAM also signals through associated intracellular proteins. The regulatory p85 subunit of phosphatidylinositol 3-kinase (PI3K) shifts its binding affinity from N-cadherin to EpCAM following overexpression of the latter [45]. The EpCAM/p85 complex displayed kinase activity, suggesting a signaling pathway of EpCAM involving activation of PI3K [45]. More recently, the embryonic Ras GTPase (ERas) was isolated as an interaction partner of EpCAM in murine teratocarcinoma cells [158]. EpCAM and ERas are expressed in pluripotent ESC and in single cells of the epiblast and visceral endoderm of murine embryos and are co-regulated at the early onset of gastrulation [158]. EpCAM/ERas interaction was associated with enhanced activating phosphorylation of AKT at serine^473^, and cellular knockout of EpCAM resulted in diminished AKT phosphorylation [158]. Furthermore, EpCAM levels regulate the activating phosphorylation of NF-κB subunit Rel A, destabilization of the inhibitor of κB (IκB), and ultimately control interleukin-8 (IL-8) expression [159]. IL-8 in turns was instrumental in the regulation of EpCAM-dependent cell invasion and positively correlated with EpCAM expression in metastatic breast cancer [159].

Thus far, we have a limited understanding of ligands that activate EpCAM signaling. EpEX is the only known extracellular ligand inducing the RIP of EpCAM [135]. Cell-cell contact likewise induced EpCAM RIP [160]. Additional extracellular cues originating from tumor cells and/or cells of the tumor microenvironment remain elusive.

### Spatiotemporal expression of EpCAM in normal tissue and carcinomas

Decades after its discovery on cancer cells, EpCAM was found to be highly expressed on murine and human ESCs [161, 162], and on murine embryonic germ cells [163]. High levels of EpCAM correlate with the pluripotent state of ESCs [137, 161, 162] and with the re-acquisition of pluripotency during reprogramming of somatic cells [138, 139, 164]. In fact, the uphold co-expression of EpCAM with reprogramming factors Nanog and Sox2 is mandatory for somatic cells to progress towards reprogrammed induced pluripotent stem cells (iPSCs) [165].

Single-cell RNA sequencing disclosed that EpCAM is expressed in cells of the inner cell mass (ICM) at gestation day E3.5 of mouse embryogenesis [158]. Epiblast, primitive and visceral endoderm, and earliest cells of the forming primitive streak express EpCAM, although to levels that are reduced compared to ICM cells. With the onset of gastrulation at E6.5, EpCAM expression was strongly reduced or absent in early mesodermal cells [158]. In *Xenopus*, EpCAM controls cell movements during embryonic development in the three germ layers [166]. Overexpression of EpCAM in a gain-of-function mutation in meso- and ectodermal cells enhanced their invasive potential and thereby induced an intermixing of the cell types based on the repression of the activity of novel protein kinase C variants [166, 167].

At later stages of murine embryogenesis (> E8.5), EpCAM expression was primarily detected in murine endodermal tissue [46, 158]. Spatiotemporal regulation of EpCAM expression was reproduced in murine ESCs after spontaneous differentiation in EBs and confirmed the lack of EpCAM in vimentin-positive mesodermal cells and its retention in Foxa2/Gata4-positive endodermal cells [158]. Balanced EpCAM expression in differentiating ESC appears mandatory as the co-existence of EpCAM-positive endodermal cells and EpCAM-negative mesodermal cells was required for full ESC differentiation [158]. Interference with this controlled expression yielded ESCs with reduced pluripotency and differentiation capacity, and with impaired capability to generate cardiomyocytes [158, 161]. Disturbance of ERas expression hampered spontaneous ESC differentiation in similar fashion [158], suggesting that EpCAM/ERas signaling is required in murine ESC for full pluripotency. The requirement of EpCAM-positive and EpCAM-negative cells for the development of contracting cardiomyocytes confirmed previous reports [168–170]. Through transcription factor Gata4, EpCAM-positive visceral and definitive endoderm is formed from pluripotent ESCs [168]. Both endoderm types support the generation of cardiomyocytes from mesodermal cells through the production of cardiac-inducing factors, and themselves differentiate towards liver progenitors [168, 169]. Accordingly, mature hepatocytes lack EpCAM, whereas human hepatic progenitors express EpCAM [171, 172] without activating it through RIP [148]. EpCAM’s role in hepatocyte differentiation was uncovered in zebrafish, where EpCAM is enriched in endodermal cells and counteracts the repression of WNT signaling to induce hepatocyte differentiation. Molecularly, EpCAM blocks Kremen-1 and Dickkopf-2 interactions through sequestration of Kremen-1, preventing Wnt receptor Lrp6 (lipoprotein receptor-related protein 6) withdrawal from the plasma membrane. As a result, Wnt2bb signaling is activated and promotes hepatocyte differentiation from endodermal cells [173].

Hence, spatiotemporal expression and functions of EpCAM during embryogenesis are tightly regulated and required for the proper development of endo- and mesodermal cells in cell-autonomous and non-autonomous ways.

### Regulation of EpCAM in cancer progression: the EMT path and metastasis formation

In line with an absence of EpCAM in mesodermal cells, frequent loss of EpCAM occurs in malignant cells undergoing EMT [174–176]. First reports of a transient EpCAM regulation in cancer progression came from xenotransplantation models. Primary tumors and larger metastases (≥ 30 cells) expressed high levels of EpCAM, whereas smaller metastases (< 15 cells) lacked EpCAM [55]. Loss of EpCAM coincided with increased expression of mesenchymal marker vimentin in primary human tumors [72, 174, 176] and in disseminated tumor cells [21, 177, 178]. Because EMT is neither an all-or-nothing process nor does it apply to the totality of malignant cells in a tumor, EMT promotes inter- and intratumor heterogeneity [74, 80, 174, 179, 180], fostering tumor progression, metastasis formation, and therapy resistance within subsets of cells [76, 174, 180, 181]. An involvement of EMT in the metastatic cascade implies the transient and reversible acquisition of migratory and invasive traits by subsets of tumor cells through a partial mesenchymal transition that can be regulated at the epigenetic level [179, 182]. Alternatively, pre-existing subsets of tumor cells in an EMT state are present at earliest stages of tumor formation and represent metastatic progenitors that can co-evolve within primary tumors [183] or at distant sites following early detachment from primary tumors [184]. It must also be noted that the actual contribution of EMT to the metastatic cascade is a matter of strong debate [179] with reports on the lack of requirement for a mesenchymal shift for the generation of distant metastases [185, 186].

Single-cell RNA-seq of oropharyngeal carcinomas uncovered a huge degree of inter- and intratumor heterogeneity at the transcriptome level [187]. A signature of partial EMT associated with metastasis formation and poor prognosis and was inversely correlated with a signature of epithelial differentiation in which EpCAM was a major determinant [187]. Functionally, tumor cells in an EMT state are more refractory to therapy and possess increased migratory and invasive traits, supporting local and distant dissemination [179, 181, 187, 188]. Eventually, metastatic outgrowth requires that disseminated tumor cells re-adopt a proliferative, epithelial phenotype that is associated with the reversion of EMT (i.e., MET). In summary, EMT emerged as a transient state with gradual changes, which strongly contributes to tumor progression and which can be monitored using a panel of dominant markers including EpCAM.

Changes along the EMT path involving loss of EpCAM are likewise observed in CTCs [177, 178] and DTCs [189]. This may have repercussions at two levels: (i) loss of EpCAM in CTCs undergoing EMT will hamper their detection using the EpCAM-based CellSearch enrichment system and (ii) it raises the question as to whether EpCAM-negative CTCs and DTCs have metastatic potential. Clinical studies on CTCs using CellSearch underscored the prognostic value of EpCAM-positive CTCs in various cancers [20, 21, 82, 84–90]. A study in castration-resistant prostate cancer and MBC patients revealed that the frequency of EpCAM-low CTCs was not correlated with the patients’ overall survival, while of EpCAM-high CTC frequency was associated with poor overall survival [190]. CTCs and DTCs with differing EMT status—as measured by EpCAM expression—were likewise detected in a mouse model of MBC [21]. EpCAM-positive CTCs with an epithelial phenotype and a restricted mesenchymal shift showed the highest metastatic potential and were associated with distant metastases and poorer survival in MBC patients [21]. In contrast, the increase of EMT-type CTCs in MBC patients correlated with disease progression following therapy [178], and mouse-derived mesenchymal-type CTCs were less susceptible to chemotherapeutic drugs *in vitro* [21]. In conclusion, a variety of EMT types of CTCs and DTCs appear to coexist in individual animals and in patients, which can be classified using EpCAM as a marker. From the most recent reports, it seems that rather epithelial-type CTCs are the source of metastases, whereas more mesenchymal-type CTCs represent treatment-resistant cells.

In addition to being a valuable marker for the EMT status of tumor cells, EpCAM is also functionally involved in the regulation of EMT. A double-negative feedback loop, in which activated extracellular regulated kinase 2 (Erk2) directly and indirectly repressed the transcription of *EPCAM* through binding to its promoter region and through the induction of EMT transcription factors that repress *EPCAM* transcription, was reported [30]. EpCAM on the other hand repressed ERK activation and thereby dampened EMT induction [30]. In nasopharyngeal carcinomas, high expression of EpCAM correlated with metastasis formation *in vitro* and *in vivo* but had no effect on cell proliferation. Strong expression of EpCAM promoted EMT and a cancer stem cell phenotype in association with increased migration and invasion, *via* the activation of AKT, mTOR, p70S6K, and 4EBP1 [191]. In colon cancer cell lines, the expression of EpCAM enhanced the transcription of reprogramming factor genes c-Myc, Oct3/4, Sox2, and Nanog, and the EMT regulators Snail and Slug through EpICD signaling [192].

Activation of EpCAM RIP through EGFR signaling was described in endometrial cells, which eventually results in the release of EpICD and the activation of EMT-relevant genes in cooperation with transcription factor LEF-1 [140]. For unknown reasons, the reported cleavage of EpCAM following EGF treatment could not be reproduced in a variety of carcinoma cell lines [143]. However, a functional connection of EpCAM and EGFR emerged very recently. We demonstrated that EGFR has a dual capacity to either induce proliferation or EMT in HNSCC cells, a phenomenon that was dependent upon the strength of activation of the downstream effector kinase Erk1/2 [143]. EpEX was revealed as a novel EGFR ligand in HNSCC [143] and in colon cancer cells [119]. EpEX induces classical EGFR-mediated pathways (i.e., AKT and Erk) but induces Erk1/2 activation to a lesser extent than EGF in HNSCC cell lines, resulting in a mild cell proliferation but no EMT. In contrast, treatment of HNSCC cell lines with EMT-inducing concentrations of EGF and equimolar amounts of EpEX blocked EMT through decreased Erk1/2 activation [143]. Patients suffering from EGFR^high^/EpCAM^low^ HNSCC were characterized by very poor survival, whereas EGFR^low^/EpCAM^high^ patients had an excellent clinical outcome [143]. Furthermore, EpEX was shown to activate EpCAM RIP *via* EGFR signaling, resulting EpICD generation [119]. EpICD was required for β-catenin accumulation in the nucleus and for activation of hypoxia-inducible factor 1 alpha (HIF1α). Accordingly, nuclear localization of EpICD in colon carcinoma patients was associated with metastasis formation and worsened clinical outcome [119]. Interestingly, anti-EpCAM monoclonal EpAb2-6 inhibited nuclear translocation of EpICD and induced apoptosis. Thus, EpAb2-6 might represent a novel and promising treatment to reduce metastasis formation in patients at risk [119].

Lastly, EpEX binding to EGFR promoted the multipotency of mesenchymal stem cells through enhancement of pluripotency factors. Mechanistically, EpEX induced an EGFR-dependent STAT3 activation and the blockade of Let7 microRNA through upregulation of LIN28 [193]. As a result, EpEX induces proliferation of bone marrow-derived mesenchymal stem cells. Hence, EpCAM regulates the fate of various stem cells through multiple mechanisms including membrane-associated signaling and as a ligand of EGFR. Furthermore, EpCAM is regulated during EMT and is itself a regulator of this trans-differentiation program in carcinoma and stem cells. Several modes of action have been reported that involve RIP products EpEX and EpICD, and full-length EpCAM. Interestingly, EMT-promoting and EMT-repressing functions of EpCAM have been described, a controversy that deserves further investigation.

## Perspective

In the future, we expect further insight in the role of EpCAM in the regulation of cell fate in health and disease, which would eventually revive its usage as therapeutic target. The discovery of RIP of EpCAM and the generation of the signaling-active fragments EpEX and EpICD paved the way for a novel class of EpCAM inhibitors that target signaling in cancer and the metastatic cascade. Antagonizing antibodies such as EpAb2-6 and small molecule inhibitors that compete with EpCAM RIP or with EpICD functions could develop into promising candidate drugs for the treatment of EpCAM-positive carcinomas and the formation of lethal metastases.

EpCAM will retain a central role as of anchor molecule in the enrichment of CTCs that harbor metastatic potential in advanced cancer patients. Here, EpCAM has dual potential as a quantifier of systemic tumor cells in the frame of liquid biopsies and as a potential target for adjuvant therapy of residual tumor cells with specific biologicals.

Clinical studies on the predictive potential of EpCAM for carcinoma entities associated with a high degree of treatment resistance are required for the use of EpCAM as biomarker in clinical decision-making. For example, prospective studies on the expression of EGFR and EpCAM might shed light on the power of EpCAM expression to predict treatment response in carcinoma entities that implement a therapy with the anti-EGFR monoclonal antibody cetuximab or small molecule inhibitors of EGFR.

In the future, the role of EpCAM in pluripotent stem cells might gain momentum. Especially, the implication of EpCAM and EpICD in the generation of iPS is of relevance. Additionally, the use of EpEX in combination with the reprogramming factors Klf4 or Oct3/4 was instrumental in the generation of iPS and might therefore experience (pre-)clinical application.

Lastly, it can be anticipated that basic research on the expression dynamics and the molecular functions of EpCAM in cancer and healthy cells will further support the abovementioned clinical applications of this highly versatile molecule.

## Conclusion

Over the past four decades, EpCAM has evolved from a humoral antigen expressed on the majority of carcinoma cells to a complex signaling molecule involved in central aspects of cell fate such as proliferation, pluripotency, differentiation, and organ integrity. Signaling by intact EpCAM and the proteolytic fragments EpEX and EpICD as well as signaling-independent functions of EpCAM serve these various purposes and represent novel handles to tackle metastatic malignancies. Furthermore, EpCAM remains an attractive target for antibody-based cancer therapies and as a biomarker for patient stratification. Importantly, EpCAM is the central target molecule for the enrichment and characterization of systemic tumor cells with prognostic and metastatic potential. Eventually, EpCAM became a multifaceted protein with a long-standing history in molecular oncology and in stem cell biology.

## Data Availability

Not applicable.

## Abbreviations

AKT: serin/threonine kinase (protein kinase B; PKB)
4EBP1: eukaryotic translation initiation 4E-binding protein 1
c-Myc: cellular Myc
CTC: circulating tumor cell
CTE: congenital tufting enteropathy
DTC: disseminated tumor cell
EGF: epidermal growth factor
EGFR: epidermal growth factor receptor
EMT: epithelial-to-mesenchymal transition
EpCAM: epithelial cell adhesion molecule
EpEX: EpCAM extracellular domain
EpICD: EpCAM intracellular domain
Erk: extracellular regulated kinase
ESC: embryonic stem cells
FHL2: four-and-a-half LIM domain 2
GTPase: guanine triphosphatase
HNSCC: head and neck squamous cell carcinoma
ICM: inner cell mass
iPS: induced pluripotent stem cells
Klf4: Krüppel-like factor 4
LEF-1: lymphoid enhancer factor 1
Let7: lethal 7
LIN28: abnormal cell lineage 28
mAb: monoclonal antibody
MBC: metastatic breast cancer
mTOR: mammalian target of rapamycin
Oct3/4: octamer-binding factor 3/4
PI3K: phosphatidylinositol 3-kinase
p70S6K: p70 S6-kinase
RIP: regulated intramembrane proteolysis
STAT3: signal transducer and activator of transcription 3
Sox2: SRY (sex-determining region Y)-box 2
